# Carbon-Fibre-Reinforced SiC Composite (C/SiSiC) as an Alternative Material for Endoprosthesis: Fabrication, Mechanical and In-Vitro Biological Properties

**DOI:** 10.3390/ma11020316

**Published:** 2018-02-22

**Authors:** Aline Reichert, Michael Seidenstuecker, Rainer Gadow, Hermann O. Mayr, Norbert P. Suedkamp, Sergio H. Latorre, Partick Weichand, Anke Bernstein

**Affiliations:** 1 Department of Orthopedics and Trauma Surgery, Medical Center—Albert-Ludwigs-University of Freiburg, Faculty of Medicine, Albert-Ludwigs-University of Freiburg, Hugstetter Straße 55, 79106 Freiburg, Germany; aline.reichert88@web.de (A.R.); hermann.mayr@uniklinik-freiburg.de (H.O.M.); norbert.suedkamp@uniklinik-freiburg.de (N.P.S.); sergio.latorre@uniklinik-freiburg.de (S.H.L.); anke.bernstein@uniklinik-freiburg.de (A.B.); 2 Institute for Manufacturing Technologies of Ceramic Components and Composites, University of Stuttgart; Allmandring 7b, 70569 Stuttgart, Germany; rainer.gadow@ifkb.uni-stuttgart.de (R.G.); patrick.weichand@ifkb.uni-stuttgart.de (P.W.)

**Keywords:** biomaterials, carbon-fibre reinforced SiC-Composites, biocompatibility, endoprosthetics, wear

## Abstract

Particle-induced periprosthetic osteolysis and subsequent aseptic implant loosening are a major cause of compromising the long-term results of total joint replacements. To date, no implant has been able to mirror radically the tribological factors (friction/lubrication/wear) of in vivo tribological pairings. Carbon-Fibre Reinforced SiC-Composites (C/SiSiC), a material primarily developed for brake technology, has the opportunity to fulfil this requirement. Until now, the material itself has not been used in medicine. The aim of this investigation was to test the suitability of C/SiSiC ceramics as a new material for bearing couples in endoprosthetics. After the preparation of the composites flexural strength was determined as well as the Young’s-modulus and the coefficient of friction. To investigate in vitro biological properties, MG 63 and primary human osteoblasts were cultured on C/SiSiC composites. To review the proliferation, the cytotoxicity standardized tests were used. The cell morphology was observed by light microscopy, ESEM, confocal and 3D-laserscanning microscopy. C/SiSiC possesses a high resistance to wear. Cells exhibited no significant alterations in morphology. Vitality was not impaired by contact with the ceramic composite. There was no higher cytotoxicity to observe. Regarding these results, C/SiSiC ceramics seem to be biologically and mechanically appropriate for orthopaedic applications.

## 1. Introduction

Biomaterials interact with human tissue and body fluids to treat, improve, or replace anatomical structures of the human body. Total replacement of large natural joints is performed 2–3 million times per year [1]. Current material standards for an artificial joint include polymers like ultrahigh molecular weight polyethylene (UHMWPE [2], metals like titanium (CP-Ti [3]), metal alloys like cobalt chromium CoCr or Ti_6_Al_4_V [4] and ceramics like alumina (Al_2_O_3_) or zirconia-toughened alumina (ZTA) [5]. Aluminium and zirconium-oxide ceramics (Al_2_O_3_, ZrO_2_) have been employed as ceramic-to-ceramic or ceramic-to-synthetic (primarily polyethylene (PE)) bearing couples. They exhibit lower abrasion values than polyethylene and metal. Moreover, their wear particles reveal less biological activity [6,7]. These ceramics offer great mechanical and chemical stability, as well as good resistance to wear. One of their drawbacks is the excessive brittleness typical of monolithic ceramics that can lead to material failure [1,8].

To date there has been no material for endoprosthetics providing excellent resistance to abrasion and corrosion combined with great tensile strength, fracture toughness and bending strength, as well as adequate biocompatibility. Resistance to wear is important to ensure the least possible release of material particles into the body. Such particles can trigger inflammation [9] and particle-induced osteolysis, which can cause the prosthesis to loosen [10,11,12].

The high demands placed on prosthetic materials in terms of their abrasion and ductility have led to the testing of the suitability of other non-oxide ceramics such as silicon nitrite (Si_3_N_4_) [13], silicon carbide (SiC) [14] or polyetheretherketone reinforced with carbon fibres (PEEK) [1,15]. These materials facilitated an improvement in mechanical properties allied with good biocompatibility [16,17]. The material class of non-oxide ceramics appears to be well suited to achieving the goal of better bearing couples.

Carbon-fibre-reinforced silicon carbide (C/C-SiC or C/SiSiC) is as a ceramic compound a potentially novel biomaterial offering higher ductility and durability than comparable oxide ceramics. The carbon fibres increase the tolerance for damage and enhance the composite material’s properties during bending stress. To ensure such damage tolerance, an adapted and relatively weak fibre-matrix-interface is needed. If the fibre and matrix are too firmly bonded, the prosthesis can become brittle and fail, as would a monolithic material. Until now, this material has been employed primarily in brake technology thanks to its resistance to wear and low density [18,19,20]. Rajzer et al. [21] reported that reinforcing SiC with carbon fibres encourages adhesion and even the growth of collagenic connective tissue on osteoblasts. C/SiSiC ceramics have not been used in medicine so far and have therefore not been subjected to biocompatibility tests. It is their low abrasion coefficient and high resistance to pressure that make C/SiSiC ceramics interesting as sliding partner.

Aim of this investigation was to test the suitability of C/SiSiC ceramics as a new material for bearing couples in endoprosthetics. One essential quality that any new material must possess is biocompatibility. For this project, the in-vitro biocompatibility was investigated by using cuboid like scaffolds made of ceramic matrix composites (CMC). To determine whether the material is suited as a lubricant partner in endoprosthetics, its abrasion coefficient and wear tolerance against various antibodies were measured.

## 2. Results

### 2.1. Characterizing the Materials

**Figure 1:**
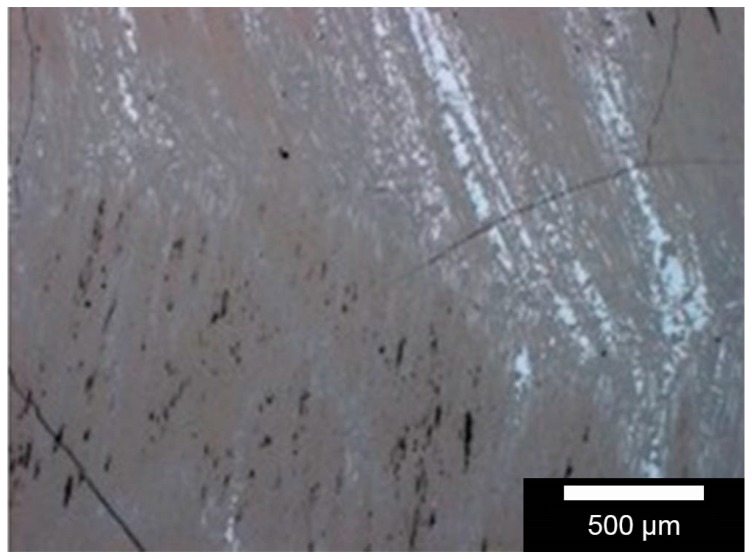
Surface-analysis of carbon fibre-reinforced silicon carbide (C/SiSiC)-cubes. White-light interferometric images 50×. Pale grey = silicon; dark grey = silicon carbide; black = carbon fibres or pores, or breaks that occurred before sample preparation.

The surface roughness of C/SiSiC was determined as arithmetical roughness average Ra-value of 0.25–0.3 µm and an average roughness depth of Rz 1.5–2 µm. The three integrated phases-silicon, silicon carbide and carbon in each of their brought-in portions are illustrated in Figure 1 in reflecting microscopic images for analysis of the C/SiSiC samples’ surface composition. On the sample surface, one finds the three phases (distributed statistically for the most part and worthwhile for later use), each in its appropriate percentage.

The proportion of half-metallic silicon that is representative of two samples is 3.04 ± 0.61 vol %. As also apparent in the reflecting microscopic images, the proportion varies from 0% to roughly 12%. The same applies to the widely differing proportions of the carbon fibres at the surface. Table 1 illustrates other material characteristics.

The samples’ porosity was detected between 1% and 3%. The flexural strength at room temperature was approx. 180 MPa, while the elongation at break was about 0.13%. The Young’s modulus was detected between 120 and 150 GPa. The density lies between 2.5 and 3.0 g/cm^3^.

**Figure 2:**
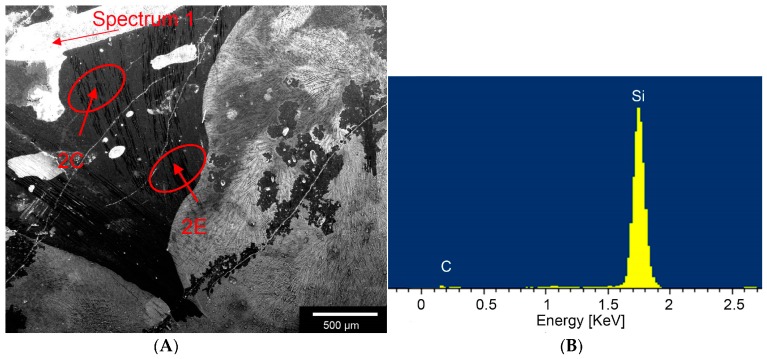

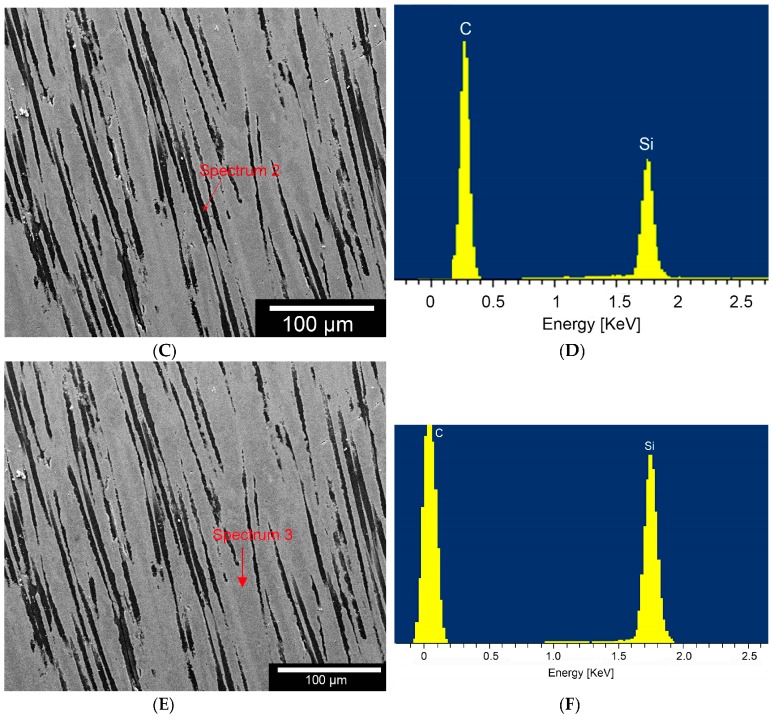
ESEM and EDX analysis of C/SiSiC-cubes, ESEM images taken with Fei Quanta 250 FEG with sensor for secondary electrodes, FEI, Hillsboro, OR, USA; (**A**): Overview of the different compositions of the sample, carbon fibres, SiC and Si; (**B**): EDX Spectrum of white area (see Figure 2A) mainly Si; (**C**): Higher magnification of Figure 2A, Arrow marks the area where the EDX spectrum (1) was taken; (**D**): EDX spectrum (2) of the Carbon Fibres; (**E**): higher magnification of Figure 2A and arrow marks where the EDX Spectrum was taken; (**F**): EDX spectrum (3) of SiC.

**Figure 3:**
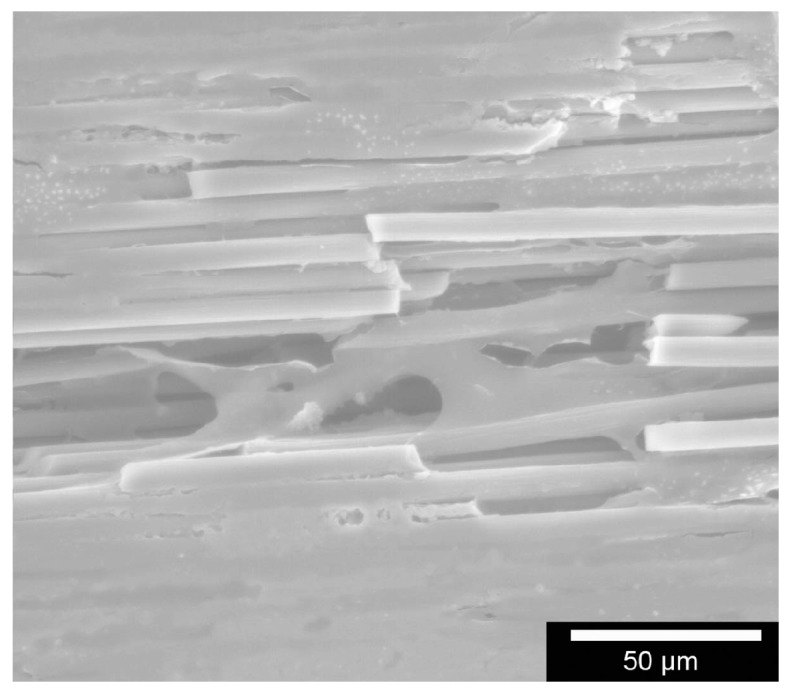
ESEM image to demonstrate morphological behaviour of cells on the sample structure. The circles in the images show cellular extension along the fibres.

Figure 2 illustrates an ESEM image involving an EDX trial revealing peaks in the silicon and carbon. This confirms different compositions within the composite. The EDX analysis shown in Table 2 revealed a 1:1 ratio of the samples with the carbon’s atomic mass measuring 45.26%, the silicon’s 54.74%. Figure 3 shows an ESEM image demonstrating morphological behaviour of cells on the sample structure. Cells were in close contact to a structure they grew onto it.

### 2.2. Friction and Wear Test

Investigating the tribological properties, a friction coefficient µ between 0.31 aluminium oxide (Al_2_O_3_) and 0.45 bearing steel (100Cr6) were detected for dry friction. The measurement of the lubricated friction properties using foetal calf serum as lubricant showed lower values on a level of 0.27 (Al_2_O_3_) and 0.41 (100Cr6).

**Figure 4:**
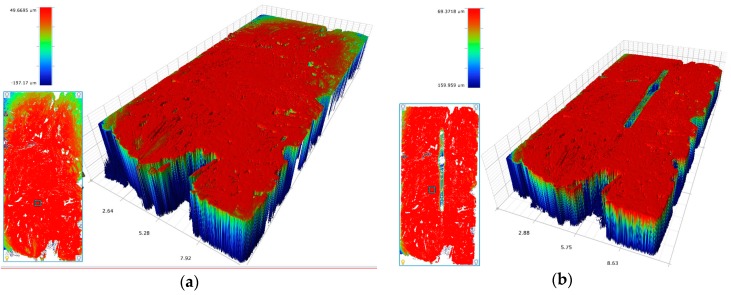
White-light interferometric images of sample surfaces before (**a**) and after (**b**) the wear mark [19].

**Figure 5:**
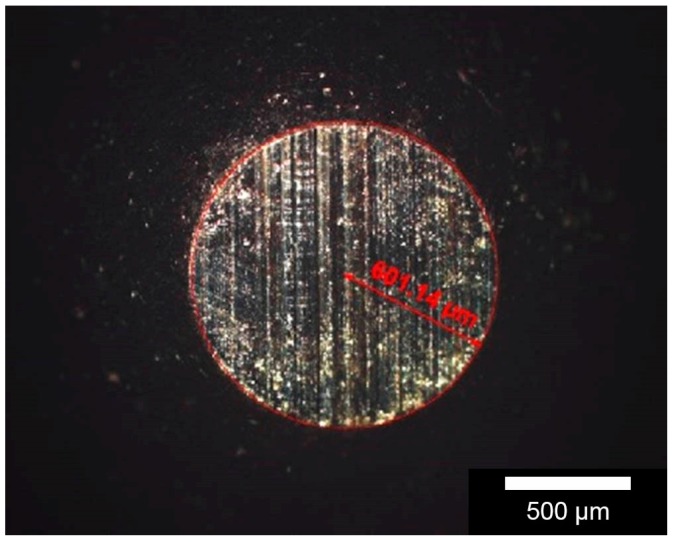
Typical surface wear pattern on a 100Cr6 ball [22]. Resulting tracks of abrasion investigated by a perthometre after the friction ball was sliding oscillative on the polished sample and the volumetric wear was determined.

Figure 4 illustrates two white-light interferometric images of the same sample slice before and after the experiment. The wear mark appears relatively even. In this example, it is about 1 mm wide and 100–150 µm deep. The behaviour of the opposed body appears quite different. While hardly any wear was detectable on the test ball made of alumina, the amount of material loss on the (100Cr6) ball is easy to measure. Figure 5 shows the respective wear scare on a 100Cr6 ball on the sample above with a volumetric wear coefficient between 0.02 and 0.44 mm³/Nm.

### 2.3. Biocompatibility

MG 63 cells and human osteoblasts were used to proof the biocompatibility of C/SiSiC composites. Therefore, metabolic activity, cell viability, cytotoxicity and cell morphology were estimated during contact with the C/SiSiC samples.

**Figure 6:**
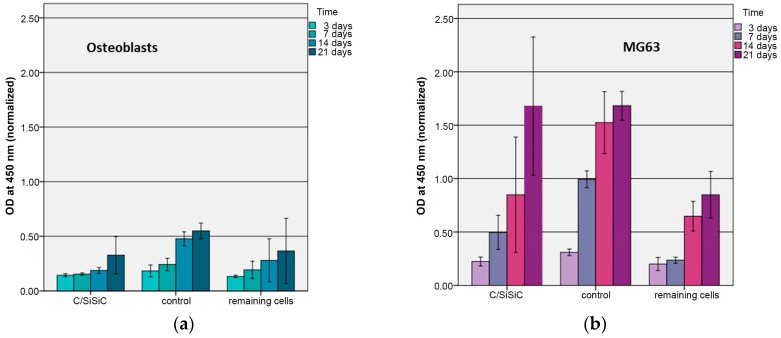
WST assay to demonstrate proliferation of human osteoblasts (**a**) and MG 63 (**b**) on the samples. C/SiSiC-cells on the composite after removing it out of the cell culture plate; control-cells only, remaining cells-cells remaining in the same cell culture plates, which were moving from the top of the composite and settled at the walls of the cell culture plate, all values were normalized to 1 cm^2^.

**Figure 7:**
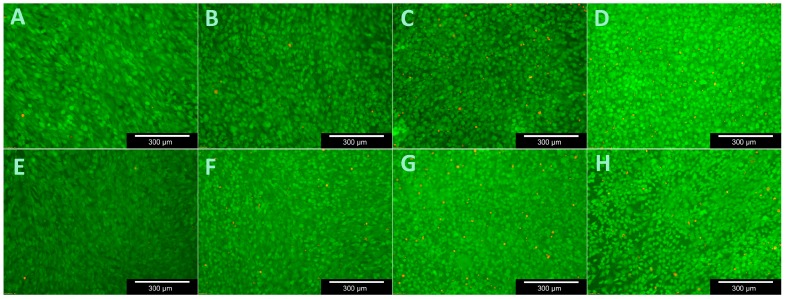
Viability assay with MG 63 for 21 days. (**A**–**D**): samples (days 3, 7, 14 and 21); (**E**–**H**): controls (days 3, 7, 14 and 21).

**Figure 8:**
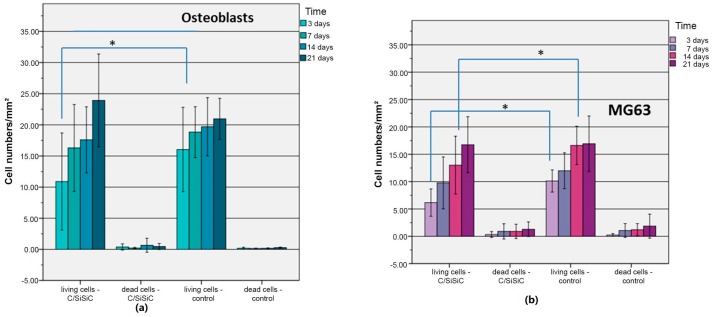
Live/dead assay: Cell number/mm^2^ (living cells and dead cells) of human osteoblasts (**a**) and of MG 63 cells (**b**) on the C/SiSiC samples. Significance set at *p* < 0.05 are assigned the same symbol *.

**Figure 9:**
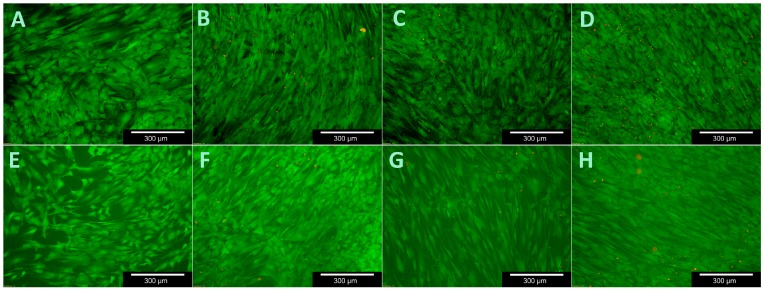
Viability assay with human osteoblasts for 21 days. (**A**–**D**): samples (days 3, 7, 14 and 21); (**E**–**H**): controls (days 3, 7, 14 and 21).

The WST-1 assay tests the respiratory chain via the mitochondrial succinate-tetrazolium-dehydro-genase system. This allow to assess the cells’ metabolic activity and is often used to investigate proliferation when testing the biocompatibility of novel materials [23,24]. Figure 6 demonstrates that both MG 63 and OB proliferated evenly on the samples. Their growth differed in terms of absolute numbers. These results are confirmed by live/dead staining (Figure 7, Figure 8 and Figure 9).

There was a significant difference in the proportion of vital cells in MG-63 after 3 (*p* = 0.01) and 14 (*p* = 0.021) days compared to the controls and a significant difference in the OB was detected after just 3 days (*p* = 0.012). Figure 7 and Figure 9 demonstrate the vitality of cells after contact with C/SiSiC over 21 days. Figure 8 shows clearly that compared to the controls, the cell proliferation through the samples was not impaired.

**Figure 10:**
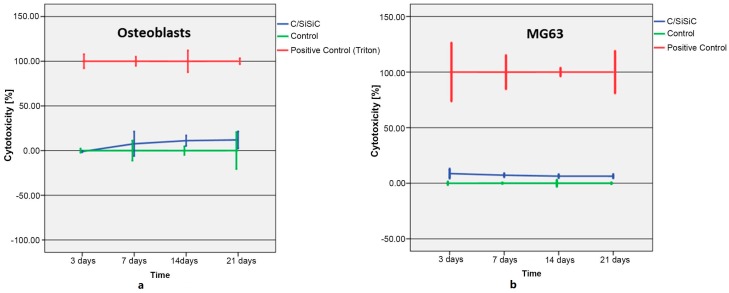
Cytotoxicity of samples versus that of human osteoblasts (**a**) and MG 63 (**b**) in per cent.

The LDH test is colorimetric and quantifies cell death and cell lysis by measuring LDH activity. Cell death is accompanied by damage to the plasma membrane, thus vital cells release little LDH into the medium. Results showed that only few cells died—which live/dead assay confirmed. A slightly higher cytotoxicity was observed compared to control group. Figure 10 shows the cytotoxicity course (%) in both cell lines after contact with C/SiSiC samples for 3, 7, 14, und 21 days. MG 63 revealed a relatively steep rise that flattened out after 14 days. The human OB exhibited a drop-in cytotoxicity over time.

**Figure 11:**
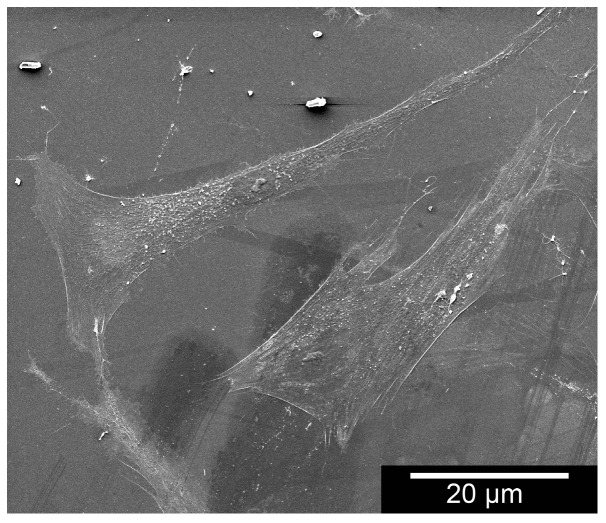
ESEM image of OB after 48 h incubation on Thermanox-membranes.

**Figure 12:**
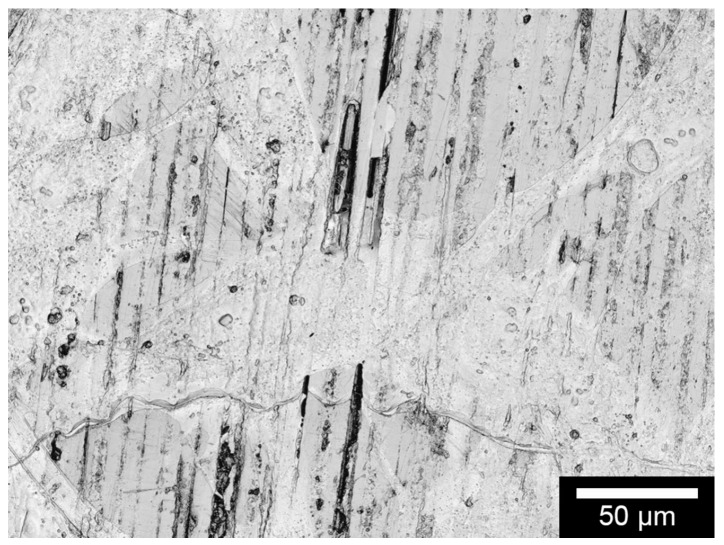
Laser scanning microscopic image of OB on samples. Laser image, images taken with Keyence VK-X 200, Magnification 1000×.

To assess the cells‘ phenotype and any alterations after having come into contact with the test material, microscopic methods like ESEM and laser scanning microscopy were used. Figure 3 shows also the cell behaviour on the samples’ structure. The cells have been coloured red via Adobe Photoshop elements 5.0 to ease interpretation. The osteoblasts and MG 63 adhered strongly to the material and exhibit no morphological anomalies. For comparison purposes: note the behaviour and phenotype of the osteoblasts on the Thermanox membrane in Figure 11. There is no perceptible morphological aberration. This is confirmed in the 3D laser scans in Figure 12, which demonstrates the behaviour of the osteoblasts on the new material’s surface in laser- and reflected-light microscopy.

## 3. Discussion

New materials and improvement of used bearing partners are very important for the development of prosthetics. To make sure that biomaterials can be set in the human body, the interaction between biological cells and the extracellular matrix must be analysed in in-vitro investigations. In this research project a completely new material was analysed because it offers the advantage of high thermic a chemical stability, enormous hardness, low tribological values, good wear resistance and improved fracture toughness because of fibre reinforcement. Aim of this research project was to test the biocompatibility of a complete new material and if this material is an improvement as bearing partner for prosthetics. The experiments showed that the mortality rate of the MG 63 and the human osteoblasts was not higher than compared to the control experiments. The phenotype and adherence of the cells on contact with the new material showed no indication for abnormal development. In addition, the material properties were investigated and compared. The tensile strength and the breaking resistance were to a considerably degree better compared to the mostly used oxide-ceramics.

Compared with the characteristics of the classic monolithic or stabilized ceramics associated with endoprosthetics such as zirconium-stabilized aluminium oxide (ZTA), our ceramics’ flexural strength values and Young’s modulus are (at least to some extent) much lower [18,25]. In terms of breaking mechanics, the CMC delivers much greater strain to rupture and a much less catastrophic failure behaviour. Moreover, CMC mechanical characteristics are more similar to the replaced parts of bones thus enabling a much more homogeneous load distribution. The relatively low Young’s modulus of C/SiSiC ceramics compared to monolithic ceramics (120–150 GPa) can be attributed to the quasi-isotropic short-fibre reinforcement in the CMC and the desirably weak interface between the fibres and the matrix, which is what enables the high elongation at break in the first place. Therefore, CMC mechanical characteristics are more similar to the replaced parts of bones thus enabling a much more homogeneous load distribution. An ideal, unidirectional fibre orientation with a strong fibre bond would theoretically amount a value of about 300 GPa correlating the rule of [26]. Our results concerning the Young’s modulus regarding each phase’s hardness concur with findings in the literature; however, there is a certain amount of minor deviation depending on the applied test force, as the elastic area’s percentage becomes more apparent in conjunction with small loads. This is also obvious when measuring carbon fibre filaments with 7 µm diameter. If the loads are too low, the Vickers indentations are too weak, whereas when the load is excessive, the indentations area extends into the matrix and you get a mixed hardness of two or more phases. What is important in this situation is silicon carbide’s extreme hardness. The literature reports a SiC hardness of HV_0.1_ = 2428 and HV_0.1_ = 1345 for silicon [18]. We confirmed those values (HV_0.1_ = 2879 ± 264 and HV_0.1_ = 1312 ± 25, respectively), with minor deviations. The carbon fibres’ value, measured at the cross-section, was HV_0.01_ = 524 ± 81, somewhat lower than otherwise-reported values reflecting the influence of the filaments’ bond on the matrix. A mixed hardness will be tribologically effective depending on the surface composition and quality, contact surface and friction partner.

The relatively low porosity values for the CMC are attributable to the moderate amount of fibres in region weight percent (wt %) and an adjusted pore network in the preforms prior to silicon infiltration.

A friction coefficient µ between 0.31 (Al_2_O_3_) and 0.45 (100Cr6) were noted—relatively high values for tribological pairing. However, those measurements were taken at relatively high surface pressure (Hertzian pressure of 10 N and 5 mm ball diameter, idealized punctual contact, approx. 530 MPa). In the field of endoprosthetics, even in a worst-case scenario (such as cushioning a jumping movement with a heavy backpack), the surface pressure will be nowhere near that intensity. Thus, lower values would be most likely. What is important in this situation is silicon carbide’s extreme hardness. One of the factors the coefficient of friction depends on is the surface’s phase composition. Silicon carbide delivers very high abrasion coefficients, whereas silicon and carbon, as well as carbon fibres, tend to yield rather low values. Moreover, this value also depends on the surface structure. With systems that operate dry, smooth surfaces are preferable. With tribological systems employing a lubricating medium, structured surfaces may be beneficial to ensure the lubricant‘s even distribution (see honing pattern in cylinder liners). The wear on the basic body is minimal regardless of its type. The evaluated values for the coefficient of friction µ are relatively high for a sliding application. Also, the use of foetal calf serum as lubricant in order to minimize these values featured no significant benefit (minus 10–14%). The serum tended to dehumidify in the testing cycle and adheres on the sliding surface. A change in the phase composition and an adapted textile structure for the carbon fibre reinforcement will significantly help to reduce the friction to a lower value. The wear behaviour of the C/SiSiC is excellent, requiring a suitable counterpart. Alumina shows good properties. Hardened steel is too weak for a long time usage. A carbon fibre reinforced SiC would be the ideal counterpart, combining damage tolerance with wear resistance.

The observed wear mechanisms can be divided into two sections. For the test set up with a 100Cr6 ball (hardness HV_1_ = 960), a significant abrasion can be detected on the ball, as well as on the sample surface. After testing, the ball shows typical abrasion wear marks due to the lower hardness of the ball compared to the hard phases of the sample (SiC and Si). The wear marks on the sample surface can be split into different aspects. The carbon fibres show a significant lower hardness compared to the 100Cr6 material and show a significant brittle failure behaviour if they are loaded traversal. This brittle behaviour and the existing porosity of the C/SiSiC generate particles of all three phases, which are sticking to the wear marks of the ball and acting as additional abrasive grain. Therefore, the hard phases SiC and Si also show a minor abrasive wear track although their hardness is superior to the 100Cr6. This can be observed in Figure 4 in an unevenly formed wear track. Additionally, a minimal amount of adhesion on the wear track can be detected due to ferritic deposits analysed via EDX analysis. No plastic deformation mechanisms were observed as the surface stays plane at the edges of the wear track on the same level.

For the test set up with the Al_2_O_3_ ball (hardness HV_1_ = 2000) a different wear mechanism can be observed. The high hardness of the ball implies nearly no detectable wear at its surface, therefore the smooth surface of the ball stays completely intact. No abrasive particles from the sample surface are quarried and no abrasive wear mechanism is observed. This can be referred also to the lower coefficient of friction of this test set up. This fact enables a different wear behaviour on the sample surface, showing a smoothening on the oscillation track. Also in this case no plastic deformation can be observed.

According to Byeong-Choon et al. we could observe different compositions, like SiC, carbon fibres or Areas with a high concentration of Si within the composite by using the EDX.

The low changes of cell vitality after contact with ceramic specimens as described in literature could be confirmed. Cappi et al. [14] already demonstrated that non-oxide ceramics led to no changes in vitality in live/dead assays in L929 murine fibroblasts and human mesenchymal stem cells. Their finding concurs with ours here.

As Markhoff et al. [23] also reported about their WST-results, the sample revealed significantly lower extinction values than the controls at each time point. When considering the cause of this superiority over the controls, one needs to keep in mind that the Thermanox membranes we used have a larger surface but we were working with the same number of cells. They could thus spread out over a broader surface and adherence persisted longer. López-Álvarez et al. [27] showed that pre-osteo-blastic MC3T3-E1 cells on biological silicon carbide grew well compared to the growth on poly-styrene (material from tissue-culture vessels). Furthermore, we know that large differences in cell proliferation on ceramics are triggered when their surfaces are modified [28].

About the cytotoxicity investigations, we need to remember again that for control purposes, the Thermanox membranes’ somewhat larger surface, was colonized by the same number of cells, which then had a larger surface onto which they could adhere. This test is often only carried out for 24 to 72 h. In many biocompatibility tests of orthopaedic materials, however, it is customary to run the tests for longer periods, as it is well known that lactate dehydrogenase secretion does not change significantly after brief testing periods [29,30]. It can, however, change considerably after longer test phases. Moreover, long-term cell contact in vivo is considerably longer, meaning that test results after 48 h deliver little useful information. The limitations of this test are the medium’s own LDH and FBS activity, necessary to cultivate the cells. To minimize this background activity, the FBS concentration was lowered to 1%, thus reducing and eventually restricting the culturing conditions for the cells.

The human OB compared to MG63 exhibited a drop-in cytotoxicity over time cells. These divergent findings can be attributed to the cell lines’ growth behaviour. The same number of cells were used, although the tumour cells have a much faster doubling time. MG 63’s very rapidly increasing cell numbers made it difficult for new cells to adhere to the samples, which in turn led to increased cell death. This difference causes the tumour cells to raise their lactate dehydrogenase level, thus this test’s indicator of cytotoxicity. MG 63’s rise in cytotoxicity on the samples over time is attributable to a high number of cells on a surface that is too small.

Similar experiments of the biocompatibility of silicon nitrite (Si_3_N_4_), another non-oxide ceramic, revealed good results in terms of cytotoxicity, proliferation and cell adhesion [13,22]. Compared with those findings, our test results demonstrate the impressive biocompatibility of non-oxide ceramics. 

In summary, carbon-fibre reinforced silicon carbide possesses a high resistance to wear and is thus a promising biomaterial for orthopaedic bearing applications in endoprosthetics. It revealed good biocompatibility in vitro.

## 4. Materials and Methods 

### 4.1. Sample Preparation

The C/SiSiC samples tested were produced via the Liquid Silicon Infiltration (LSI) of pyrolized porous fibre preforms made by warm-flow pressing free-flowing granulates on a hydraulic downstroking press with a heated die of the type HPS-S, 1000 kN (Hydrap-Schuler, Goeppingen, Germany). Sample dimensions are 20 mm × 20 mm × 10 mm. The granulates were prepared via granulation in an intensive mixer Type R02 (Maschinenfabrik Gustav Eirich GmbH & Co KG, Hardheim, Germany). The mixtures contain the following ingredients: carbon short fibres 3 and 6 mm, HT (Toho Tenax GmbH, Wuppertal, Germany); SiC-Powder, SM03 (Industriekeramik Hochrhein, Wutöschingen, Germany), Polyethylene-granulate, (Dow Chemical Company, Midland, MI, USA), phenolic resin 6227FP (Bakelit AG, Erkner, Germany), distilled water and ethanol. After crosslinking in the heated die, one obtains a polymer-matrix composite reinforced with short fibres (PMC), which then transforms thermally into a porous carbon fibre reinforced carbon composite under an inert gas atmosphere (CFC or C/C). Pyrolysis usually occurs at 900 to 1000 °C for 2–10 h under nitrogen. After pyrolysis, the now-porous fibre preforms containing carbon are infiltrated by molten half-metallic silicon in a high-temperature vacuum oven Type HTK80 (Carbolite Gero GmbH & Co. KG, Neuhausen, Germany). During the pore scaffold’s infiltration process (vacuum and capillary-aided), the liquid silicon reacts with the excess carbon released from the pyrolyzed phenolic resin and turns into silicon carbide. This procedure is carried out at temperatures between silicon’s melting temperature (approx. 1414 °C) and 1850 °C—usually between 1550 and 1700 °C [31]. This process requires a minimal oversupply of silicon, so that there are three to four phases in the CMC: carbon fibres, silicon and silicon carbide (reaction-sintered and from the filler material) and under certain conditions, some residual carbon. After the siliconization process, the samples are ground and polished (using a diamond suspension down to a particle size of 1 µm) and cut into 1 cm^3^ pieces for cell culture experiments.

The sample bodies were cleaned after the experiments with sodium hypochlorite (2.5%) and repeatedly washed in twice-distilled water for 15 min and immersed in an ultrasonic bath. Later they were washed in 30% ethanol for 15 min in an ultrasonic bath and then again for 15 min twice in distilled water. The sample bodies were then autoclaved at 121 °C [27]. 

### 4.2. Sample Characterisation

Phase distribution on the sample surface was determined by microscopic images, taken with a MeF4 reflected light microscope (Leica Microsystems GmbH, Wetzlar, Germany). These measurements are taken at several locations to account for the standard deviation associated with this process. The different grey values represent the different compositions of the composite.

Individual phases were demarcated using Vickers-micro-hardness measurements with a Fischerscope HCU (Helmut Fischer GmbH, Sindelfingen, Germany). As the phases were so finely distributed and partially brittle, we applied two different test forces: HV_0.01_ (0.09807 N) and HV_0.1_ (0.9807 N).

The samples’ porosity was determined by using a mercury porosimeter Pascal 140-240/440 (Porotec GmbH, Hofheim am Taunus, Germany). 

Two procedures were carried out to assess the mechanical characteristics. Flexural strength was determined at room temperature according to DIN658-3 as three-point-bending with a universal testing machine Z100 (Zwick GmbH & Co. KG, Ulm, Germany). Sample dimension for mechanical characterization was 200 mm × 20 mm × 10 mm. Testing speed was 2 mm/min. The support clearance was calculated according to Equation (1).

(1) l=20×h

with

l: Support clearance (mm)
h: Thickness of the sample (mm)

The calculation of the flexural strength was performed in accordance to Equation (2).

(2) $$\sigma_{f,m}=\frac{3F_{m}}{2bh^{2}}$$

with

$\sigma_{f,m}$: Flexural strength (MPa)
$F_{m}$: Bending force (N)
b: Width of the sample (mm)
h: Thickness of the sample (mm)

The Young’s-modulus was carried out via resonant frequency-damping analysis RFDA (IMCE, Genk, Belgium).

The surface morphology of the specimens was examined using scanning electron microscopy Leo 438 VP (Carl Zeiss Microscopy GmbH, Oberkochen, Germany) with attached EDX unit.

The measurement of the surface roughness of the prepared samples was also performed on a Profilometer Perthometer PGK (Perthen-Mahr GmbH, Goettingen, Germany).

Density was evaluated by Archimedes principle on an encapsulated scale with measurement frame (Sartorius AG, Goettingen, Germany) according to DIN EN 993-1.

### 4.3. Friction and Wear Test

After preparation of the composites, the tribological characteristics were determined. The coefficient of friction µ was obtained using the oscillations-pin-on-disc tribometer, RUMED Type (Rubarth Apparate GmbH, Laatzen, Germany), measured in the oscillation mode that corresponds to the real movement course in endoprosthetics. The coefficient of friction results from the two factors friction force FR and normal force FN. 80000 oscillations (each 20 mm of travel, oscillating at 10 mm/s) at FN 10 N and 22 °C. Relative humidity was adjusted to 40%. Two different materials as, friction ball 5 mm (100Cr6-HV_0.1_ = 960 and Al_2_O_3_-HV_1_ = 2000) were selected as the test parameters. Noting the Hertzian pressure of 10 N and 5 mm ball diameter, idealized punctual contact, the approximate value for the surface pressure was 530 MPa. Additionally, the evaluation was performed in dry and in lubricated conditions. As Lubricant foetal calf serum was used. The resulting tracks of abrasion were investigated with a Profilometer Perthometer PGK (Perthen-Mahr GmbH, Goettingen, Germany) and white-light interferometry (Bruker Corporation, Billerica, MA, USA). The friction pin or ball carries out an oscillative sliding friction movement on the grounds and polished sample and the volumetric wear was normalized to one Nm (wear coefficient). To assess the phase composition, measurements with an energy-dispersive X-ray spectroscope (EDX) were taken. The wear rate of the specimens was defined by the ratio of wear volume to the normal load multiplied by the total reciprocating sliding distance. All the friction and wear tests were performed with three samples of each type and the average values are presented hereafter. Prior to tribological tests, the specimens were cleaned up in deionized water to eliminate dust and solid contamination, afterwards washed with ethanol and dried at 60 °C for 6 h.

### 4.4. Cell Culture

For the in vitro tests, a cell line and human osteoblasts were used. The use of human osteoblasts was approved by the local Ethics Committee of the University Medicine Freiburg (registration number: 305/10). The human osteoblast-like cell line MG-63 (ATCC CRL-1427) was cultured in Dulbecco’s Modified Eagles Medium: Ham’s F12 (DMEM) (Gibco, Invitrogen™, Grand Island, New York, NY, USA) with 10% foetal bovine serum (FBS) (Biochrom AG, Berlin, Germany), penicillin (100 U/mL)/streptomycin (100 U/mL) (P/S) (Gibco, Invitrogen™, Grand Island, New York, NY, USA) at 37 °C and 5% CO_2_. Primary cultures of human osteoblast like cells (OB) were prepared from the bone specimens as previously described [32]. The femoral heads of patients undergoing primary hip replacement because of primary osteoarthritis were collected. In brief, trabecular fragments from the proximal femur were cut into pieces, thoroughly rinsed in phosphate-buffered saline (PBS; 18 mM CaC1_2_, 0.2 mM MgC1_2_) and kept in M199 (Gibco, Invitrogen™, Grand Island, New York, NY, USA) with l-Glutamine (0.68 mM), 10% FBS, penicillin (100 U/mL), streptomycin (100 U/mL) and gentamycin (50 U/mL) at 37 °C and 5% CO_2_. Cultures were initiated within three hours and fed twice weekly. These cells were shown to produce alkaline phosphatase and collagen I. Cells of passage 2 were used. A separate primary culture for each donor was set up. The cells were counted in a haemocytometer and their viability was determined using trypan blue (GIBCO, Invitrogen™, Grand Island, New York, NY, USA). After having achieved a confluence of about 80%, the cells were sub cultivated in trypsine (Gibco, Invitrogen™, Grand Island, New York, NY, USA). For the live/dead assay, the WST assay to assess cytotoxicity and adherence, three of the 1-cm³ sample bodies were placed in a 24-well-plate (Costar microtiter plates, Corning Incorporated, New York, NY, USA). The test samples were seeded directly with 10^5^ cells in 100 µL medium. After an adhesion period of two hours, the well was filled with 1.2 mL culture medium from each cell. How recommended in manual to do a substance control for the LDH-Assay one more scaffold was used to compare the reaction between scaffold and medium without cells. To monitor the material effect, the same procedure was repeated with the same number of cells on Thermanox^®^ membranes (Thermo Scientific, Waltham, MA, USA). All tests were repeated three times.

### 4.5. Cell Biological Testing

#### 4.5.1. Metabolic Activity

The metabolic activity of MG-63 cells and OB were determined after 3, 7, 14 and 21 days via mitochondrial succinate-dehydrogenase activity (WST-1-test) (Roche Diagnostics GmbH, Mannheim, Germany). The conversion of tetracolium salt to coloured formazan by mitochondrial succinate dehydrogenase from metabolically active cells was measured spectrophotometrically using an ELISA reader (Magellan Measurement Parameter Editor infinite M200, Tecan, Salzburg, Austria) at 450 nm. For each day three scaffolds were examined. The medium was removed from the microtiter plates and the sample bodies were transferred to a new 24-well-plate. The old and new plates were filled with 1.2 mL phenol red-free medium and 120 µL of WST-stock solution was added. After a minute on the shaker, they were incubated for 4 h at 37 °C and 5% CO_2_. The plate was then shaken again for another minute and 100 µL of each sample was then transferred to a new 96-well-plate. The adsorption, which was directly proportional to the metabolic cell activity, was measured. The medium in the plates that were not to be tested was always exchanged on the test days. The assay was repeated three times.

#### 4.5.2. Viability

To test cell viability during contact with the C/SiSiC samples, the live/dead cell staining Kit II (Roche Diagnostics GmbH, Mannheim, Germany) was used. It allows the vital cells to be stained green using calcein AM and the dead cells red using ethidium homodimer III (EthD-III) together with an effective plasma membrane. The standard work solution (2 µM Calcein AM, 4 µM EthD-III) was continuously refreshed prior to light-microscopic assessments with DPBS.

Cell viability was analysed after 3, 7, 14 and 21 days by replacing the medium from the microtiter plates that were not being assessed. Three cuboid-like scaffolds for each test were used. The medium was first removed from those experimental plates and the sample forms were then purified with DPSB. Thereafter the surfaces of the samples were moistened with 150 µL of standard work solution and incubated for 10 min at room temperature. The ceramic cubes were then evaluated under the fluorescence microscope (Olympus BX51, Tokyo, Japan) and photographed slice-by-slice. Ten images were taken of various areas on each sample’s surface at 5-, 10- and 20-times magnification for future assessment.

#### 4.5.3. Cytotoxicity

The cytotoxicity of C/SiSiC samples on MG-63 and OB were analysed using the Cytotoxicity Detection Kit (Roche Diagnostics GmbH, Mannheim, Germany). It relies on the fact that the plasma membrane of dead or damaged cells was no longer intact and that cytoplasmatic enzyme lactate dehydrogenase (LDH) was thereby released. This kit‘s reaction reflects a redox reaction between NAD^+^ to NADH/H^+^ and lactate to pyruvate with LDH as the catalysator. In the second step, the NADH/H^+^ hydrogen atoms enable the reduction from tetracolium salt (bright yellow) to formazan (red), which is then measured spectrophotometrically. The test reagent is produced from a diaphorase/NAD^+^ mixture and iodine tetracolium chloride with sodium lactate at a 1:45 ratio. The medium was removed 24 h after seeding and 1.2 mL phenol red-free medium with only 1% FBS was added. FBS contained variable amounts of LDH, which can lead to increased background absorption: likewise, phenol-derivatives can induce the release of LDH, thus affecting the results. Therefore, phenol red-free medium and 1% FBS was chosen to work with. A background control was also carried out to test the assay medium’s LDH activity; for the positive control, we lysed the cells with Triton-X, thereby measuring maximum enzyme activity. To evaluate the substance control there was one cuboid like scaffold without cells only with medium in one well. In total 4 cubes were used- three for the experimental sample, one for the substance sample.

Cytotoxicity was assessed on days 3, 7, 14, und 21. The medium in the remaining microtiter plates was always completely refreshed on the test days. The assay was three times conducted. On each test day, the 24-well-plate (Costar Microtiter plates, Corning Incorporated, New York, NY, USA) was centrifuged at 250× *g* for 10 min. 100 µL of the overlap was then transferred to a 96-well-plate and 100 µL of the test reagent added. This was followed by 30 min incubation in the dark at room temperature. Last of all, we measured the absorption at 490 nm in the ELISA-Reader (Magellan Measurement Parameter Editor infinite M200, Tecan, Salzburg, Austria).

#### 4.5.4. Morphology

The samples’ surface as well as cell adhesion and cell morphology were assessed via ESEM. To observe cell behaviour along the fibre structures, the sample forms were moistened in a 24-well-plate with 10^5^ cells in 100 µL medium. After two hours of incubation, the well was filled up with 1.2 mL of culture medium of that particular cell type. To investigate after 3, 7, 14 and 21 days, we cooled down the well-plate for 30 min to 4 °C and then removed the medium. Fixation took place using a 1-mL mixture of 1.5% glutaraldehyde and 0.1 M cacodylate at a pH of 7.4 for 12 h at 4 °C. The samples were then cleaned three times with Dulbecco’s Phosphate Buffered Saline (DPSB) (Gibco, Invitrogen™, Grand Island, New York, NY, USA) and osmicated for 1 h with 1% osmium tetroxide(OsO_4_). After they were washed three times in twice-distilled water, they were dehydrated in 30-, 50-, 70-, 80-, 90- and 100-% ethanol, each for three minutes [15]. After air-drying, the samples were fixed onto the sample holder and depending on their image quality, spluttered with gold. The test was repeated three times.

3D laser scanning microscopy enables us to determine the height profile, textural qualities and morphology of cells on ceramic. Ours was a 3D laser scanning microscope, VK-X200 (Keyence, Osaka, Japan). The samples were prepared for the ESEM imaging and fixated but without using osmium tetroxide. Images were taken once with laser illumination, under reflecting-light microscopy, which enabled us to calculate the height profiles.

#### 4.5.5. Statistical Analyses

The collected data were analysed descriptively using SPSS statistics software (Version 22, IBM, Ehningen, Germany). Based on the raw data the mean value and the standard deviation were calculated. The Mann-Whitney-U-Test was used to evaluate the differences between experiment and control samples. *p*-values < 0.05 were considered to indicate statistical significance.

## 5. Conclusions

In this investigation, we succeeded in demonstrating that, thanks to its mechanical properties, flexural strength (180 MPa) and strain to rupture (0.13%), carbon-fibre reinforced silicon carbide possesses a high resistance to wear (0.0 cm^3^, HV_0.1_ SiC: 2428, HV_0.1_ Si: 1345, HV_0.1_ C: 70) and is thus a promising biomaterial for orthopaedic bearing applications in endoprosthetics. It revealed good biocompatibility in vitro. The cell lines exhibited no morphological alterations and adhered well to the C/SiSiC samples. Vitality was not impaired by contact with the ceramic composite. Cell growth was observed evenly distributed over a 21-day period. Further research efforts must be made in material science to achieve lower abrasion coefficients. Recent research results in this field have already revealed considerable progress. In the future, investigators aiming to apply this composite in endoprosthetics will have to focus on its efficacy in conjunction with sudden, strong demands and long-term performance in bodily fluids within joint simulators, etc. In conclusion: C/SiSiC can definitely be considered a new material with genuine potential for use in endoprosthetics. 

## Figures and Tables

**Table 1:** Material properties of C/SiSiC.

**Porosity**	1–3%
**Bending Strength**	ca. 180 MPa
**Ultimate Strain**	ca. 0.13%
**Density**	3.0 g/cm³
**E-Modul**	35% C-fibre: 230 GPa
	2–5% Si: 130 GPa
	2–5% rest carbon: ca. 80 GPa
	(35 × 230 + 5 × 130 + 5 × 80 + 55 × 380)/100 = 300 GPa
	Hypothetical with 100% bonding connection and complete
	unidirectional fibre arrangement → brittleness
	In fact, ca. 120–150 GPa
**HV_0.1_ SiC**	2428
**HV_0.1_ Si**	1345
**HV_0.1_ C**	70

**Table 2:** Spectrum characteristics of energy-dispersive X-ray spectriscopy.

EDX Spectra	Element	Mass (%)	σ Mass (%)	Atom (%)
Spectrum 1	C K	7.98	9.78	16.89
	Si K	90.08	9.58	81.49
	Na K	0.61	0.20	0.67
	Cl K	1.33	0.30	0.95
Spectrum 2	C K	91.34	0.22	96.20
	Si K	8.12	0.19	3.66
	Ti K	0.54	0.09	0.14
Spectrum 3	C K	26.13	3.03	45.26
	Si K	73.87	3.03	54.74
